# Anesthesia Services in Low- and Middle-Income Countries: The Fragile Point for Safe Surgery and Patient Safety

**DOI:** 10.7759/cureus.43174

**Published:** 2023-08-08

**Authors:** Imran A Khan, Habib Md R Karim

**Affiliations:** 1 Community and Family Medicine, Baba Raghav Das Medical College, Gorakhpur, IND; 2 Anesthesiology, Critical Care, and Pain Medicine, All India Institute of Medical Sciences, Deoghar, Deoghar, IND

**Keywords:** low- to middle-income countries, challenges faced, health services administration, perioperative anesthesia service, safe surgery, patient health safety

## Abstract

The Safe Surgery Saves Life campaign of the World Health Organization advocates patient safety best practices during surgical procedures. Anesthesia service is indivisible from the patient safety best practices. Although anesthesia services are safer than ever before, safe delivery of anesthesia service and patient safety depends significantly on the availability of qualified anesthesiologists, the knowledge and competency of anesthesiologists, the work environment, and the availability of essential equipment and monitoring facilities. Despite anesthesiologists being the midstream of perioperative care, their role and service are often underacknowledged, especially in low- and middle-income countries (LMICs). Anesthesia services in LMICs face myriad challenges such as a shortage of skilled personnel, inadequate resources, limited training opportunities, and minimal administrative say, which act as the fragile point in the chain of safe surgery delivery. Specific solutions should focus on strengthening the anesthesia workforce, providing fair remuneration and incentives, advocating for anesthesia autonomy, and facilitating access to educational resources. Nevertheless, managing these problems requires a collaborative effort involving governments, healthcare organizations, and international stakeholders to develop sustainable solutions and prioritize the well-being of both anesthesia providers and patients. This editorial focuses on it briefly, emphasizing the anesthesia of rural healthcare service and patient safety.

## Editorial

Much of the world’s population needs better access to timely, safe, and affordable surgical care. The Safe Surgery program of the World Health Organization (WHO) has one such motto to improve healthcare and reduce perioperative morbidity and mortality. As the world strives for it, an often-overlooked aspect is the sustained availability and quality of anesthesia services. A recent survey found 550,134 specialist/physician anesthesiologists around the globe. Low-income and lower-middle-income countries (LMICs) represent only 15% of this workforce [1]. In many LMICs, including India, the shortage of skilled anesthesiologists and inadequate resources present significant challenges in providing safe anesthesia care for surgical procedures, interventional practice, and many obstetric cases. In this editorial, issues encountered by anesthesiologists in various areas are examined, and the urgent need for specific actions to improve the anesthesia workforce is highlighted to enhance patient care and the provision of Universal Health Coverage.

Challenges

Professionals

The significant scarcity of anesthesia professionals is one of the most urgent problems in LMICs. As evidenced by a cross-sectional survey conducted in East Africa, the density of anesthesiologists per 100,000 population in these countries is remarkably low, ranging from 0.02 in Burundi to 0.39 in Kenya [2]. The World Federation of Societies of Anesthesiologists reports that rural and isolated locations with limited access to surgical treatment are mainly suffering from a scarcity of anesthesia professionals [3]. A similar situation exists in India, where the ratio of anesthesiologists to population is notably inadequate. This scarcity of anesthesia providers leads to the deprivation of safe anesthesia and surgery for a substantial proportion of the population. Existing professionals routinely deal with severe clinical loads and few resources, leading to burnout and negatively affecting mental and physical well-being.

Anesthesia Equipment and Monitoring Devices

Many healthcare institutions, especially those in rural areas and smaller private hospitals, lack the essential and emergency monitoring tools, medications, and other supplies needed to provide safe anesthesia services [4]. This equipment scarcity can significantly impact the quality of anesthesia care, potentially negatively influencing patient outcomes.

Training and Educational Resources

To successfully manage complicated and high-risk patients, anesthesiologists must be competent. The education and professional development of anesthesia providers might differ greatly among LMICs. Although some anesthesiologists may have received extensive training, others may have only had access to limited resources for skill development. Anesthesiologists must have a comprehensive knowledge base and the right attitude toward patient care to provide safe anesthesia [5]. Inadequate training may result in compromised patient safety and fatal outcomes.

Service Acknowledgment and Remuneration Disparities

Anesthesiologists in LMICs like India often struggle with low remuneration, and often anesthesiologists are underpaid compared to their counterparts. This disparity in payment often results in financial strain for anesthesiologists, making it challenging to maintain a sustainable practice. Moreover, low remuneration can also lead to decreased motivation and job satisfaction, potentially impacting the quality of care they provide and adopting some alternative fields of practice for earning their bread and butter.

In LMICs, including India, anesthesiologists often depend on surgical consultants for work opportunities. Surgeons typically decide which anesthesiologist to call for a surgical procedure, creating a reliance on these consultants for consistent work. This dependence and lack of autonomy for anesthesiologists might limit their ability to negotiate better service terms and remuneration. Additionally, it could create a hierarchical structure where anesthesiologists’ opinions are not sufficiently considered during decision-making, impeding improvements in anesthesia practices. Violence against doctors and other medical personnel has increased considerably in the present era, and serious attention to tackling the situation is the need of the hour for adequate healthcare delivery [6].

Many hospitals in LMICs encounter significant disparities in accessing quality anesthesia equipment [4], leading to compromised patient care. One of the primary factors contributing to the disparity in anesthesia equipment is the financial limitations faced by the establishment of LMICs. More funds are needed to purchase modern anesthesia machines, patient monitors, and equipment. As a result, anesthesia services often use outdated or poorly maintained devices, jeopardizing patient safety and overall healthcare quality [7]. The recent economic crisis in Sri Lanka also showed the impact on safety and quality; drug and instrument re-uses became usual [8]. A center in Cambodia retrospectively evaluated the challenges and noted that lack of commonly used drugs, equipment, and monitoring were major threats to safe anesthesia delivery [9]. Anesthesia departments should be more frequently addressed in the resource allocation decisions made by hospital administrations in LMICs. Lack of support from hospital leadership and inadequate opportunities for professional development can lead to a cycle of neglect and discrimination toward the anesthesia department [10]. Administrators may prioritize other departments or medical specialties, resulting in a need for more attention and support for anesthesia services. This neglect further exacerbates the disparity and leaves anesthesia teams needing help to provide safe and effective care [11].

Way forward

While the anesthesia service-related challenges in LMICs are multitudinous, specific solutions that can help improve working conditions and provide safe anesthesia service for safe surgery. Effective teamwork and collaboration among team members are essential in the surgical ecosystem. Anesthesia providers must coordinate with other healthcare professionals to deliver comprehensive and safe care [10]. Addressing burnout and promoting resilience among healthcare professionals are crucial to maintaining a sustainable workforce. Implementing policies, including improving working conditions, delivering psychological support, and providing chances for professional growth, is necessary to maintain a resilient anesthesia workforce.

To ensure professional growth and enhance the competency of anesthesia care providers, Continuing Medical Education (CME) opportunities should be made more accessible to anesthesiologists. Governments and healthcare institutions should organize regular CME workshops, conferences, and online courses. Cooperation with international anesthesia organizations can also facilitate knowledge sharing and skill development.

Addressing the issue of low remuneration is crucial to attract and retain skilled anesthesiologists. Governments and private healthcare institutions should consider revising payment structures to ensure fair and competitive service remuneration. Furthermore, introducing performance-based incentives, professional development allowances, and improved working conditions can also enhance job satisfaction and motivate anesthesiologists to provide unhindered services.

Anesthesiologists should advocate for greater autonomy and involvement in decision-making processes. Collaboration with healthcare administrators and surgical consultants can help bridge communication gaps and empower anesthesiologists to play a more significant role in patient care planning. Anesthesiology associations and professional societies should play an active role in raising their voice for the rights and interests of anesthesiologists. Moreover, international cooperation and support are necessary to establish sustainable solutions. Collaborative efforts with organizations such as the WHO can help develop guidelines and protocols for safe anesthesia care in resource-limited settings. Additionally, initiatives like the Lancet Commission on Global Surgery’s target of 20 surgical, anesthetic, and obstetric physicians per 100,000 population by 2030 necessitate increased funding for training physician anesthetists [12].

In LMICs, where public healthcare resources are often limited and the demand for healthcare services is high, small nursing homes often serve as the primary contact point for healthcare. However, these setups deal with various inherent workplace issues, which can have a negative impact on the kind of care they provide. Their capacity to offer patients the best care may need to be improved by issues such as a lack of funds, skilled healthcare workers, necessary medical equipment, and inadequate infrastructure. Governments and policymakers are crucial in formulating and enforcing standards, guidelines, and policies that healthcare providers offer. The WHO reports and guidelines can serve as valuable resources [13].

Professional societies like the Indian Society of Anesthesiologists (ISA) also play crucial roles, and the Private Practitioner Forum (PPF) of the society is a vibrant platform created to bring together anesthesiologists working in private practice throughout India while acknowledging the distinct possibilities and problems these professionals confront. The PPF works to improve the standards of private practice anesthesiology by utilizing its members’ combined knowledge and experiences, ultimately heading toward safe anesthesia practices. In addition to knowledge sharing, the PPF think tank advises on policy amendments [14]. India, with the largest poor population and relatively poor health infrastructure in rural setups, deserves special mention. While professional societies like ISA, the Government of India, and international collaborations have initiated steps to support patient safety initiatives, the initiatives still have miles to go.

The WHO Surgical Safety Checklist is essential for ensuring patient safety during surgical procedures [15]. While a comprehensive checklist outlines crucial steps for surgical teams to follow before, during, and after surgery, with specific emphasis on anesthesia machine checks, pulse oximetry, and management of difficult airways, unfortunately, accessibility to well-maintained anesthesia machines and other equipment remains limited in many LMICs due to financial constraints and infrastructure challenges [16]. When functioning correctly, the anesthesia machine delivers precise and regulated anesthetic agents. Relying solely on pulse oximetry may not be sufficient to detect all potential risks. End-tidal carbon dioxide (EtCO_2_) monitoring should be an integral part of anesthesia protocols, as it provides valuable insights into a patient’s ventilation status and helps identify complications such as hypoventilation and esophageal intubation [17]. In LMICs, access to advanced medical equipment, including EtCO_2_ monitors, remains challenging. Limited healthcare budgets and the absence of robust supply chains hinder the acquisition of necessary monitoring devices.

To address these challenges effectively, a multifaceted approach is required. Governments and healthcare organizations must prioritize and invest in strengthening the anesthesia workforce. Expanding the pool of qualified anesthesia providers allows more patients to access safe anesthesia services, particularly in underserved regions. Enhancing training programs and competency assessments will improve the quality of care delivered.

To conclude, anesthesia services in LMICs face significant challenges in delivering safe and quality service for safe surgery. Myriad challenges such as shortage of skilled personnel, inadequate resources, limited training opportunities, and minimal administrative say act as the fragile point in the chain of safe surgery delivery. While anesthesia service stands on a thin line between life and death, the crucial role of such service is belittled and underacknowledged. To address these issues, specific solutions should focus on strengthening the anesthesia workforce, providing fair remuneration and incentives, advocating for anesthesia autonomy, and facilitating access to CME. Nevertheless, managing these problems requires a collaborative effort involving governments, healthcare organizations, and international stakeholders to develop sustainable solutions and prioritize the well-being of both anesthesia providers and patients.
